# Evaluating Large Language Models for the National Premedical Exam in India: Comparative Analysis of GPT-3.5, GPT-4, and Bard

**DOI:** 10.2196/51523

**Published:** 2024-02-21

**Authors:** Faiza Farhat, Beenish Moalla Chaudhry, Mohammad Nadeem, Shahab Saquib Sohail, Dag Øivind Madsen

**Affiliations:** 1 Department of Zoology Aligarh Muslim University Aligarh India; 2 School of Computing and Informatics The University of Louisiana Lafayette, LA United States; 3 Department of Computer Science Aligarh Muslim University Aligarh India; 4 School of Computing Science and Engineering VIT Bhopal University Sehore India; 5 School of Business University of South-Eastern Norway Hønefoss Norway

**Keywords:** accuracy, AI model, artificial intelligence, Bard, ChatGPT, educational task, GPT-4, Generative Pre-trained Transformers, large language models, medical education, medical exam, natural language processing, performance, premedical exams, suitability

## Abstract

**Background:**

Large language models (LLMs) have revolutionized natural language processing with their ability to generate human-like text through extensive training on large data sets. These models, including Generative Pre-trained Transformers (GPT)-3.5 (OpenAI), GPT-4 (OpenAI), and Bard (Google LLC), find applications beyond natural language processing, attracting interest from academia and industry. Students are actively leveraging LLMs to enhance learning experiences and prepare for high-stakes exams, such as the National Eligibility cum Entrance Test (NEET) in India.

**Objective:**

This comparative analysis aims to evaluate the performance of GPT-3.5, GPT-4, and Bard in answering NEET-2023 questions.

**Methods:**

In this paper, we evaluated the performance of the 3 mainstream LLMs, namely GPT-3.5, GPT-4, and Google Bard, in answering questions related to the NEET-2023 exam. The questions of the NEET were provided to these artificial intelligence models, and the responses were recorded and compared against the correct answers from the official answer key. Consensus was used to evaluate the performance of all 3 models.

**Results:**

It was evident that GPT-4 passed the entrance test with flying colors (300/700, 42.9%), showcasing exceptional performance. On the other hand, GPT-3.5 managed to meet the qualifying criteria, but with a substantially lower score (145/700, 20.7%). However, Bard (115/700, 16.4%) failed to meet the qualifying criteria and did not pass the test. GPT-4 demonstrated consistent superiority over Bard and GPT-3.5 in all 3 subjects. Specifically, GPT-4 achieved accuracy rates of 73% (29/40) in physics, 44% (16/36) in chemistry, and 51% (50/99) in biology. Conversely, GPT-3.5 attained an accuracy rate of 45% (18/40) in physics, 33% (13/26) in chemistry, and 34% (34/99) in biology. The accuracy consensus metric showed that the matching responses between GPT-4 and Bard, as well as GPT-4 and GPT-3.5, had higher incidences of being correct, at 0.56 and 0.57, respectively, compared to the matching responses between Bard and GPT-3.5, which stood at 0.42. When all 3 models were considered together, their matching responses reached the highest accuracy consensus of 0.59.

**Conclusions:**

The study’s findings provide valuable insights into the performance of GPT-3.5, GPT-4, and Bard in answering NEET-2023 questions. GPT-4 emerged as the most accurate model, highlighting its potential for educational applications. Cross-checking responses across models may result in confusion as the compared models (as duos or a trio) tend to agree on only a little over half of the correct responses. Using GPT-4 as one of the compared models will result in higher accuracy consensus. The results underscore the suitability of LLMs for high-stakes exams and their positive impact on education. Additionally, the study establishes a benchmark for evaluating and enhancing LLMs’ performance in educational tasks, promoting responsible and informed use of these models in diverse learning environments.

## Introduction

Large language models (LLMs) are potent natural language processing tools, excelling in a range of artificial intelligence (AI) tasks, from news writing to product descriptions. They have garnered widespread attention across academia and industry [1,2], going beyond the scope of natural language processing into tasks related to health care [3], neuroscience [4], philosophy [5], marketing and finance [6,7], sociology [8], education, and others [9,10]. The development of LLMs and chatbots is experiencing an upsurge, with established companies and emerging start-ups actively engaged in their creation [11], catering to general or specific purposes [12]. Prominent examples include Generative Pre-trained Transformers (GPT)-3.5 (OpenAI), GPT-4 (OpenAI), and Bard (Google LLC) [13,14]. Other notable examples are BlenderBot, Galactica, LLaMA (FAIR) [15], Alpaca (Stanford), BloombergGPT [16], Chinchilla (DeepMind), and PaLM [17], heralding the emergence of even more chatbots in the future [12].

The public release of ChatGPT in November 2022 and Bard in March 2023 has garnered significant attention due to their general purpose and flexible nature. ChatGPT [18], built on the GPT-3.5 architecture, has become popular for its remarkable ability to generate coherent and human-like responses. GPT-4.0 represents the latest iteration, incorporating enhanced language generation and improved multiturn conversation handling. Both GPT-3.5 and GPT-4.0 have been specifically trained to interact with users in a conversational manner, maintaining context, handling follow-up questions, and even correcting themselves. Bard, on the other hand, leverages Google’s LaMDA [19], enabling it to handle a diverse range of language-related tasks and provide in-depth information.

In educational settings, students are using LLMs such as Bard, GPT-3.5, and GPT-4 to enrich their daily learning experiences [20,21]. They aid students in test preparation, offer research assistance, and contribute to their overall performance improvement and knowledge acquisition [22]. It has been observed that LLMs, despite their impressive performance, can sometimes generate text that includes fabricated or incorrect information [13,23]. Consequently, researchers have directed their attention toward investigating the test-taking capabilities of different LLMs. Numerous research studies have delved into the assessment of GPT-3.5’s efficacy in multiple-choice exams in higher education domains [24]. Some investigations have specifically focused on ChatGPT’s test-taking performance in diverse professional fields, including business [25], accounting [26], law [27], and medicine [28]. In the medical realm, authors in Bommineni et al [29] examined its competence in tackling the Medical College Admissions Test, which serves as a prerequisite for admission to most medical schools in the United States. In Gilson et al [30] and Kung et al [31], authors have scrutinized ChatGPT’s aptitude in the United States Medical Licensing Examination (USMLE), while Teebagy et al [32] conducted a comparative study of GPT-3.5 and GPT-4’s performance in the Ophthalmic Knowledge Assessment Program exam. Additionally, Ali et al [33] undertook a comparison of GPT-3.5, GPT-4, and Google Bard, using questions specifically prepared for neurosurgery oral board examinations. Similarly, Zhu et al [28] investigated ChatGPT’s performance in several medical topics, namely, the American Heart Association, advanced cardiovascular life support, and basic life support exams.

Despite the successful integration of LLMs in educational environments, a crucial question remains: can LLMs provide the necessary accuracy and reliability required for critical assessments? The published studies predominantly focus on specialized fields within medicine, with few investigations addressing the effectiveness of AI tools for medical school entrance examinations [29]. Additionally, such comparisons made in the literature typically revolve around the performance of a solitary LLM against human abilities [24,34], with limited exploration of how they compare against other LLMs or baseline models, which could provide valuable insights into the strengths and weaknesses of different LLMs. Our primary objective is to bridge this knowledge gap by undertaking a comparative analysis of 3 notable chatbots: GPT-3.5, GPT-4, and Bard, for a standardized medical school exam known as the National Eligibility cum Entrance Test (NEET).

NEET [35] is a competitive entrance exam in India for Bachelor of Medicine and Bachelor of Dental Surgery programs in both government and private colleges. Introduced in 2013 by the Medical Council of India, NEET replaced various state-level and institution-specific tests to standardize medical admissions. Since 2019, the National Testing Agency (NTA) has been responsible for conducting and supervising the NEET. The exam comprises a total of 200 multiple-choice questions aimed at testing knowledge, understanding, and aptitude in 4 subjects: physics, chemistry, botany, and zoology. Candidates can only attempt a maximum of 45 questions per subject, for a total of 180 out of 200 questions. Correct answers are awarded 4 points, while each incorrect response leads to a 1-point deduction. Candidates are allotted 3 hours to complete the examination. To qualify for admission to a medical school, candidates must obtain a minimum or cutoff score, which can change year by year. The cutoff score for NEET-2023 was 137 out of 720. In 2023, over 2.03 million students took the NEET exam [24], a number that has been rising annually by 10% to 16.5%, highlighting the exam’s widespread popularity and importance. Among the 1.15 million candidates who qualified in 2023, only 2 scored full marks (720/720), only 1 scored 716 out of 720, a total of 17 scored 715 out of 720, and 6 scored 711 out of 720 [36]. NEET’s rigorous nature, coupled with its widespread adoption, underscores its importance as the primary evaluation tool for determining students’ knowledge, aptitude, and readiness for pursuing medical and dental education at the undergraduate level [35].

In this investigation, to evaluate the performance of the 3 mainstream LLMs, namely GPT-3.5, GPT-4, and Google Bard, in answering questions related to the NEET 2023 exam, we used rigorous statistical analyses. We scrutinized each model’s performance across 3 pivotal frameworks: overall comparison, subject-level comparison, and topic-level comparison. The outcomes of this study can help premed students make informed decisions about incorporating LLMs into their test preparation strategies. To the best of our knowledge, this marks the first endeavor to undertake such a study.

## Methods

### Question Set Selection and Preparation

In this paper, we tested the performance of the 3 LLMs on NEET-2023, which was obtained as a portable document file. Although the exam consists of 200 questions, due to the presence of illustrations and diagrams, it was not possible to process all the questions. As a result, we excluded questions with illustrations, resulting in a set of 175 questions for this study. This sample size is large enough to statistically justify each model’s performance on the entire exam, with a 95% CI and a 5% margin of error. The selected questions were then manually presented to Bard, GPT-3.5, and GPT-4, and the responses were documented in Excel (Microsoft Corporation).

### Data Analysis

We compared responses generated by each model against the correct answers from the official answer key on the NEET website. Based on this comparison, the responses were either marked as correct (1) or incorrect (0).

#### Prediction Performance

Excel’s built-in functionalities were then used to generate the following comparison metrics to assess predictive performance of the LLMs:

1. Accuracy is defined as the percentage of correct responses obtained by a model. In the context of this research, accuracy was obtained using the formula:
  *Accuracy = Correct Responses / Total Responses*
2. Accuracy consensus is defined as the ratio between correct answers upon which the compared models agree to all the answers (correct and incorrect) upon which the compared models agree. The formula is
  *Accuracy consensus = Correct Responses / Total Consensus*

#### Scoring Performance

Next, we calculated the overall, subject-level, and topic-level percentage scores for each LLM following the NTA’s scoring rules. Each correct answer was awarded 4 points, while each incorrect answer resulted in a deduction of 1 point. We merged zoology and botany into a single biology category, as the topic-level analysis included questions from both fields. The overall score percentage for each model was determined by dividing the total points scored by the maximum possible points, which was 700. Subject-level percentages were derived by dividing each model’s total points by the maximum points available in that subject. Similarly, topic-level percentages were calculated by dividing the total points scored in each topic by the maximum points available for that topic, which varied across different topics.

## Results

### Prediction Performance

The results demonstrated that GPT-4 had higher accuracy and consensus compared to GPT-3.5 and Bard. It also consistently outperformed the other models across subjects and topics. GPT-3.5 and Bard showed variations in their performances, with specific strengths in certain subjects and topics.

#### Overall Accuracy

The overall accuracy rates of the models were as follows:

1. GPT-4 achieved the highest accuracy rate of approximately 54.3% by correctly identifying 95 out of 175 responses.
2. GPT-3.5 demonstrated an accuracy of 36.7%, with 64 out of 175 correct responses.
3. Bard achieved the lowest accuracy of approximately 33.1%, based on 58 out of 175 correct answers.

#### Subject-Level Accuracy

Table 1 presents the number of correct responses obtained by each model in each of the 3 subject areas covered by NEET. It was evident that GPT-4 is consistently more accurate than both Bard and GPT-3.5 in all 3 subjects. For each subject, the number of correct responses obtained by GPT-3.5 and Bard differed by ±3, indicating relatively similar subject-level accuracy rates. On the other hand, GPT-4 was substantially more accurate than the other models, generating 4 to 16 more correct answers per subject. In physics, GPT-4 achieved 73% (29/40) accuracy, followed by GPT-3.5 with 45% (18/40), and Bard with 38% (15/40). Similarly, in chemistry, GPT-4’s accuracy rate was 44% (16/36), while GPT-3.5 and Bard achieved an accuracy rate of 33% (12/36). Shifting to biology, GPT-4 maintained its lead with 51% (50/99) accuracy, followed by GPT-3.5 with 34% (34/99), and then Bard with 31% (31/99).

**Table 1:** Number of correct responses (n) and accuracy rates in each subject per model.

Subject	GPT^a^-4, n (%)	GPT-3.5, n (%)	Bard, n (%)
Biology (n=99)	50 (51)	34 (34)	31 (31)
Chemistry (n=36)	16 (44)	12 (33)	12 (33)
Physics (n=40)	29 (73)	18 (45)	15 (38)

^a^GPT: Generative Pre-trained Transformers.

#### Topic-Level Accuracy

Table 2 displays the number of correct responses obtained from each model on various topics. GPT-4 was the most accurate in 9 (50%) out of 18 topics. Moreover, for at least half (2-4) of the topics in each subject, GPT-4 demonstrated the highest accuracy. GPT-3.5 was the most accurate (8/15, 53%) in inorganic chemistry. In addition, it was more accurate than Bard in 7 topics across the 3 subjects. However, it had a 0% accuracy in population and ecology (biology) and simple harmonic motion and waves (physics). Bard was the most accurate in the topics on plant kingdom and ecosystem and environment issues. Furthermore, it was more accurate than GPT-3.5 in 5 topics across all 3 subjects. However, it has a 0% accuracy for 2 physics topics, namely modern physics and electronics and optics. GPT-4 and GPT-3.5 had similar accuracies in 1 physics topic (modern physics and electronics: 2/4, 50%) and 2 biology topics (cell biology and genetics: 7/16, 44%; and ecosystem and environmental issues: 2/5, 40%). GPT-4 and Bard are 100% accurate in the topics on simple harmonic motion and waves. All 3 models were at the same level of accuracy in the topics on biomolecules and heat and thermodynamics.

In a nutshell, GPT-4 had a higher accuracy across a wide range of topics (15/18, 83%), while GPT-3.5’s and Bard’s accuracies were well below GPT-4’s. Moreover, they showed variations in their accuracies across topics.

**Table 2:** Number of correct responses for each topic per model.

Topic	GPT^a^-4, n (%)	GPT-3.5, n (%)	Bard, n (%)
Biotechnology (n=11)	7 (64)^b^	6 (55)	4 (36)
Evolution and health (n=9)	7 (78)^b^	4 (44)	2 (22)
Population and ecology (n=6)	1 (17)^b^	0 (0)	1 (17)^b^
Biomolecules (n=3)	1 (33)	1 (33)	1 (33)
Cell biology and genetics (n=16)	7 (44)^b^	7 (44)^b^	3 (19)
Ecosystem and environmental issues (n=5)	2 (40)	2 (40)	3 (60)^b^
Plant kingdom (n=25)	8 (32)	6 (24)	11 (44)^b^
Animal kingdom (n=24)	17 (71)^b^	8 (33)	6 (25)
Physical chemistry (n=12)	6 (50)^b^	3 (25)	4 (33)
Organic chemistry (n=9)	3 (33)^b^	1 (11)	2 (22)
Inorganic chemistry (n=15)	7 (47)	8 (53)^b^	6 (40)
Mechanics (n=12)	8 (67)^b^	6 (50)	6 (50)
Heat and thermodynamics (n=3)	1 (33)	1 (33)	1 (33)
Electrostatics and electricity (n=11)	10 (91)^b^	5 (45)	6 (55)
Optics (n=3)	3 (100)^b^	2 (67)	0 (0)
Simple harmonic motion and waves (n=1)	1 (100)^b^	0 (0)	1 (100)^b^
Magnetism (n=6)	4 (67)^b^	2 (33)	1 (17)
Modern physics and electronics (n=4)	2 (50)^b^	2 (50)^b^	0 (0)

^a^ GPT: Generative Pre-trained Transformers.

^b^Highest accuracy within a topic.

### Accuracy Consensus

#### Overall Accuracy Consensus

The accuracy consensus for the pairs were approximately as follows:

1. Bard and GPT-3.5 were correct on 29 out of 69 matching responses, giving the pair an accuracy consensus of 0.42 and an accuracy of 29 (16.6%) out of 175.
2. Bard and GPT-4 were correct on 42 out of 75 matching responses, resulting in an accuracy consensus of 0.56 and an accuracy of 42 (24%) out of 175.
3. GPT-3.5 and GPT-4 were correct on 45 out of 79 matching responses, giving the pair an accuracy consensus of 0.57 and an accuracy of 45 (25.7%) out of 175.
4. All 3 models were correct on 29 out of 49 matched responses. The accuracy consensus of the trio was approximately 0.59 and an accuracy of 29 (16.6%) out of 175.

This ascending trend in accuracy consensus indicated that GPT-4 enhanced the agreement on correct responses, especially when used in conjunction with either Bard or GPT-3.5. The best accuracy consensus and accuracy were obtained when GPT-3.5 and GPT-4 were considered together. Moreover, the collective intelligence of these models was as good as the weakest duo, that is, Bard and GPT-3.5 combined.

#### Subject-Level Accuracy Consensus

Table 3 shows the total number of correct matching responses and accuracy consensus at the subject level for each model.

**Table 3:** Subject-level total correct matching responses and accuracy consensus across compared models.

Subject	GPT^a^-3.5 vs Bard		Bard vs GPT-4		GPT-3.5 vs GPT-4		Bard, GPT-3.5, and GPT-4	
	Total correct matching responses, n	Accuracy consensus	Total correct matching responses, n	Accuracy consensus	Total correct matching responses, n	Accuracy consensus	Total correct matching responses, n	Accuracy consensus
Biology	17	0.4	22	0.46	23	0.48^b^	17	0.52
Chemistry	4	0.31	7	0.50	8	0.50^b^	4	0.50
Physics	8	0.58	13	1.00	14	0.93^b^	8	1.00

^a^GPT: Generative Pre-trained Transformers.

^b^Highest accuracy within a subject.

The subject-level accuracy consensus revealed following insights.

For biology, the highest accuracy consensus was observed between GPT-3.5 and GPT-4 (n=23, ratio of 0.48), indicating GPT-4’s superior performance. This duo also produced the highest accuracy, that is, 23 (23%) out of 99. Even though the accuracy consensus of the trio was the highest, it did not correspond to the highest accuracy (17/99, 17%).

For chemistry, both comparisons involving GPT-4 (Bard vs GPT-4 and GPT-3.5 vs GPT-4) yielded a higher accuracy consensus ratio of 0.50. However, the duo of GPT-3.5 and GPT-4 resulted in highest accuracy, that is, 8 (22%) out of 36.

For physics, Bard versus GPT-4 and the collective comparison of all models achieved a perfect accuracy consensus of 1.00 and an accuracy of 13 (32%) out of 40. However, the highest accuracy (14/40, 35%) was shown by GPT-3.5 versus GPT-4, with comparable accuracy consensus of 0.93.

These points demonstrate GPT-4’s dominance across subjects, with physics showcasing the highest consensus scores. This suggests that when GPT-4 is used in tandem with any other model, the duo or trio will corroborate each other's responses more than when Bard and GPT-3.5 are considered together.

#### Topic-Level Accuracy Consensus

Table 4 shows the total number of correct matching responses and accuracy consensus at the topic level for each model.

The following observations can be made about data presented in Table 4.

GPT-3.5 versus GPT-4 demonstrated the highest accuracy consensus and number of correct matching responses in 11 (61%) out of 18 topics. This trend was followed by the Bard versus GPT-4 duo, which showed the highest number of accurate responses and accuracy consensus in 7 (39%) out of 18 topics.

“Biomolecules,” “heat and thermodynamics,” “optics,” and “simple harmonic motion and waves” had low or zero accuracy consensus for all or most comparisons.

Hence, the combined intelligence of the models cannot help with the preparation of all the topics, if the goal is to seek consensus or confirmation of responses across models.

**Table 4:** Topic-level correct matching responses and accuracy consensus across compared models.

Topic	GPT^a^-3.5 vs Bard			Bard vs GPT-4			GPT-3.5 vs GPT-4			Bard, GPT-3.5, and GPT-4	
	Total correct matching responses, n	Accuracy consensus	Total correct matching responses, n		Accuracy consensus	Total correct matching responses, n		Accuracy consensus	Total correct matching responses, n		Accuracy consensus
Biotechnology	3	0.75	3		0.60	4		0.80^b^	3		0.75
Evolution and health	3	0.75^b^	3		0.50	3		0.75^b^	3		0.75^b^
Population and ecology	2	0.67	2		0.67	3		1.00^b^	2		1.00
Biomolecules	0	N/A^c^	0		N/A	0		N/A	0		N/A
Cell biology and genetics	3	0.30	3		0.43^b^	4		0.36^b^	3		0.43^b^
Ecosystem and environmental issues	1	0.33	2		0.67^b^	1		0.33	1		0.50
Plant kingdom	2	0.22	4		0.31^b^	3		0.30	2		0.33
Animal kingdom	3	0.38	5		0.50^b^	5		0.50^b^	3		0.43
Physical chemistry	2	0.67	3		0.50	4		0.67^b^	2		0.67
Organic chemistry	1	0.50	3		0.75^b^	1		0.33	1		1.00
Inorganic chemistry	1	0.13	1		0.25	3		0.43^b^	1		0.25
Mechanics	2	0.50	3		1.00	4		1.00^b^	2		1.00
Heat and thermodynamics	0	N/A	0		N/A	0		N/A	0		N/A
Electrostatics and electricity	3	0.60	5		1.00	6		1.00^b^	3		1.00
Optics	0	N/A	0		N/A	1		1.00^b^	0		N/A
Simple harmonic motion and waves	0	N/A	0		N/A	0		N/A	0		N/A
Magnetism	2	0.50	2		1.00^b^	2		1.00^b^	2		1.00^b^
Modern physics and electronics	1	1.00	3		1.00^b^	1		1.00	1		1.00

^a^GPT: Generative Pre-trained Transformers.

^b^Highest combination of accurate responses and accuracy consensus in a topic.

^c^N/A: not applicable.

### Scoring Performance

#### Overall Scores

GPT-4 achieved the highest score with 300 (42.9%) out of 700 points, outperforming GPT-3.5, which scored 145 (20.7%) out of 700 points, and Bard, which obtained 115 (16.4%) out of 700 points. To qualify for the NEET-2023 entrance test, candidates needed to secure at least 137 out of 720 points, which represents 19.6% of the total points. It was evident that GPT-4 passed the entrance test with flying colors, showcasing exceptional performance. On the other hand, GPT-3.5 managed to meet the qualifying criteria, but with a substantially lower score. However, Bard failed to meet the qualifying criteria and, hence, did not pass the test.

#### Subject-Level Scores

The subject-level scores, as per NEET’s grading rubric, are detailed in Table 5. GPT-4 achieved the highest overall score of 42.9% (300/700), outperforming both GPT-3.5 (145/700, 20.7%) and Bard (115/700, 16.4%). In all 3 subjects, GPT-4 obtained the highest scores. GPT-3.5 scored higher than Bard in biology and physics but tied with Bard in chemistry.

**Table 5:** Subject and topic level scores for Bard, Generative Pre-trained Transformers (GPT)-3.5, and GPT-4.

Subject and topic		Scores obtained		
		Bard	GPT-3.5	GPT-4
Overall (n=700), n (%)		115 (16.4%)^a^	145 (20.7%)	300 (42.9%)^b^
**Biology (n=396)**				
	Overall	56^a^	71	151^b^
	Animal kingdom	6^a^	16	61^b^
	Plant kingdom	30^b^	5^a^	15
	Ecosystem and environmental issues	10^b^	5	5
	Cell biology and genetics	–1^a^	19^b^	19^b^
	Biomolecules	2^b^	2^b^	2^b^
	Population and ecology	–1^b^	–6^a^	–1^b^
	Evolution and health	1^a^	11	26^b^
	Biotechnology	9^a^	19	24^b^
**Chemistry (n=160)**				
	Overall	24	24	44^b^
	Inorganic chemistry	15^a^	25^b^	20
	Organic chemistry	1	–4^a^	6^b^
	Physical chemistry	8	3^a^	18^b^
**Physics (n=144)**				
	Overall	35^a^	50	105^b^
	Modern physics and electronics	–4^a^	6^b^	6^b^
	Magnetism	–1^a^	4	14^b^
	Simple harmonic motion and waves	4^b^	–1^a^	4^b^
	Optics	–3^a^	7	12^b^
	Electrostatics and electricity	19	14^a^	39^b^
	Heat and thermodynamics	2^b^	2^b^	2^b^
	Mechanics	18	18	28^b^

^a^Lowest scorer within the topic.

^b^Top scorer within the topic.

We then analyzed the breakdown of the total scores obtained by Bard, GPT-3.5, and GPT-4, categorized by subject. Of the total GPT-4 score, 50.3% (151/300) came from biology, 35% (105/300) came from physics, and 14.7% (44/300) came from chemistry. For GPT-3.5, biology contributed 49% (71/145) of the score, physics contributed 34.5% (50/145), and chemistry contributed 16.6% (24/145). Lastly, Bard’s score breakdown showed that 48.7% (56/115) from biology, 30.4% (35/115) came from physics, and 20.9% (24/115) came from chemistry.

These results show that GPT-4 outperformed both GPT-3.5 and Bard in the NEET grading rubric, achieving the highest overall score and the top scores in each individual subject. While GPT-3.5 demonstrated better performance than Bard in biology and physics, it tied with Bard in chemistry. The breakdown of scores by subject revealed that for all 3 models, the largest portion of their scores came from biology (understandably, because there were twice as many questions in this category), followed by physics, and then chemistry, indicating a consistent pattern in their relative strengths across these subjects.

#### Topic-Level Scores

The results in Table 5 shows that GPT-4 exhibited strong performance across all topics in physics but showed a relative weakness in inorganic chemistry within the chemistry subject. Bard, compared to the GPT versions, excelled specifically in the biology topics of the plant kingdom and ecosystem and environmental issues. Both GPT models performed equally well in cell biology and genetics (biology) and in modern physics and electronics (physics). Additionally, GPT-3.5 stood out for its excellent performance in inorganic chemistry, highlighting its strength in this area of the chemistry subject.

## Discussion

### Overview

We evaluated the decision-making performance of 3 models—Bard, GPT, and GPT-4—using accuracy, accuracy consensus, and test scores for the NEET-2023 entrance test. Subject-wise and topic-wise analyses were also conducted. GPT-4 consistently outperformed Bard and GPT across all subjects, achieving the highest accuracy rates: 73% (29/40) in physics, 44% (16/36) in chemistry, and 51% (50/99) in biology. Topic-wise comparisons also demonstrated GPT-4’s excellence in 15 (79%) out of 19 topics, with Bard and GPT excelling in certain topics. Particularly, Bard excelled in simple harmonic motion and waves, while GPT showed strength in inorganic chemistry. Overall, GPT-4 emerged as the top performer, excelling in both subjects and specific topics. Our findings are in line with previous studies that have also examined how LLMs perform on exams related to medical education. Bommineni et al [29] found that GPT-3.5 performs at or above the median performance of the Medical College Admissions Test takers. Ali et al [33] reported that GPT-4 outperformed both GPT-3.5 and Bard by achieving the highest score of 82.6% in specialized questions prepared for neurosurgery oral board examinations. Friederichs et al [34] found that GPT-3.5 answered about two-thirds of the multiple-choice questions correctly and outperformed nearly all medical students in years 1-3 of their studies. Gilson et al [30] reported that GPT-3.5’s performance on the USMLE was either at or near the minimum passing threshold, even without domain-specific fine-tuning. Below, we present both practical and research implications of our findings to enrich the existing literature.

### Implications

#### Practical Implications

The findings have important implications for users who need to select a model based on specific requirements and their desired score. The subject- and topic-level scores highlight the suitability of different models for different domains. GPT-4 appears to have the highest score (300/700, 42.9%), followed by GPT-3.5 (145/700, 20.7%), and then Bard (115/700, 16.4%). This demonstrates that Bard was not able to pass the NEET-2023 admission exam, and GPT-3.5 was only 2% (14/700) away from the cutoff score, which is 19% (133/700).

Although GPT-4 appears to be the preferred choice for NEET preparation, it is important to note that GPT-4 is a subscription-based service and the pricing model is uniform across the globe, which makes this model less accessible to the general audience in some parts of the world, particularly low-income countries. When cost is an issue, prospective medical school students might consider using GPT-3.5 and Bard in tandem to develop specialized knowledge and expertise in specific subject topics. The accuracy consensus metric demonstrates that the duo was correct on 29 (42%) out of 69 matching responses, reaching 16.6% (29/175) overall accuracy. However, this duo did not excel in any of the subjects, compared to the other duos. Moreover, at the topic level, it only excelled in “evolution and health.” These results suggest that, in the absence of GPT-4, while students may consider both GPT-3.5 and Bard together for exam preparation, due to the low level of consensus between these models, the total score would still fall below the cutoff score. Moreover, students would be more often confused about the correct responses while cross-checking answers with these models. Therefore, it is recommended that, for exam preparation, students do not solely rely on these models or model duos; instead, they should consult primary sources in conjunction with these models.

#### Research Implications

While scoring performance comparisons help us evaluate whether these models are able to ace the NEET-2023 exam or not, prediction performance comparisons help us evaluate their long-term performance beyond NEET 2023. The models’ predictive accuracy rates match their scoring performance. GPT-4 demonstrated the highest accuracy rate among the 3 models, indicating its superior capability to provide correct responses and its reliability as an accurate study partner. However, there is still plenty of room for improvement since its accuracy was only at 54.3% (95/175), suggesting that anyone using this model for exam preparation would be exposed to a little over 50% (100/200) of accurate information. GPT-3.5 (64/175, 37.6%) and Bard (58/175, 33.1%) had similar overall accuracy rates that are much lower compared to GPT-4’s, suggesting that these 2 models would require significant fine-tuning to qualify as reliable study aids for NEET.

The subject- and topic-level accuracy comparisons highlight specific areas where these models could benefit from domain-specific enhancements. GPT-4 demonstrated superior accuracy across all 3 subjects and 15 topics but required further improvements in 3 topics, that is, ecosystem and environmental issues, plant kingdom, and inorganic chemistry. GPT-4 excelled in at least 1 topic from each subject category, including simple harmonic motion and waves and optics in physics, physical chemistry in chemistry, and evolution and biotechnology in biology. Bard excelled in simple harmonic motion and waves, and GPT-3.5 notably excelled in inorganic chemistry. GPT-3.5, besides requiring improvements in its overall prediction capabilities, needs to develop predictive expertise in population and ecology (biology) and simple harmonic motion and waves (physics). Similarly, Bard needs to develop predictive capabilities in modern physics and electronics and optics, in addition to requiring substantial enhancements in its overall predictive capabilities.

In summary, the implications and applications of this study on LLM and education are far-reaching. First, it could serve as a benchmark for evaluating and improving LLMs’ performance in exams and other educational tasks, enhancing the overall effectiveness of these models in educational settings. Second, the use of LLMs as tutors, mentors, or peers has the potential to significantly enhance students’ learning outcomes and motivation, particularly in a country such as India with a vast student population and diverse learning needs. Last, this approach could serve as a platform to explore and address ethical and social concerns related to LLMs in education, such as issues of fairness, bias, privacy, and accountability, ensuring responsible and informed use of these models in educational contexts.

### Limitations and Further Research

Similar to any other research, this study has certain limitations that should be considered carefully. It is important to note that this study did not involve direct input from actual students, teachers, or medical school boards to understand their perspectives on these mainstream LLMs’ capability to answer questions on basic science concepts. Moreover, we do not know how prospective examinees are using these models for exam preparation or whether they trust them for critical issues such as exam preparation.

LLMs have evolved considerably just in the last 6 months. Therefore, the results of this study will have to be revisited at a later stage. For example, it is possible (and likely) that the relative performance of the different models will change over time. While Bard is currently lagging GPT-3.5 in this area, improvements to the model could mean that it might catch up to GPT-3.5 in the future. Since there is currently an “AI race” among many technology firms, it is only a matter of time before new models are introduced that could perform better on these types of questions.

### Conclusion

In this study, we conducted a comparative analysis of 3 notable chatbots, Bard, GPT-3.5, and GPT-4, to evaluate their performance on NEET-2023, a highly competitive medical school entrance examination in India. The study involved the preparation of NEET-2023 questions for the chatbots, data collection, data analysis, and scoring performance assessments.

Our results indicate that GPT-4 not only passed the NEET-2023 entrance test with a score of 42.9% (300/700) but also demonstrated higher accuracy and consensus compared to both GPT-3.5 and Bard. Particularly, GPT-4 consistently outperformed the other models across subjects and topics, achieving an overall accuracy of approximately 54.3% (95/175). GPT-3.5 and Bard, on the other hand, showed variations in their performances, with specific strengths in certain subjects and topics. Regarding subject-wise scoring, GPT-4 excelled in physics and biology while Bard performed well in chemistry.

These findings shed light on the proficiency of LLMs in answering high-stakes examination questions, particularly in the context of medical entrance exams such as the NEET. GPT-4’s superior performance and accuracy suggest its potential utility as a valuable resource for medical students seeking assistance in test preparation and knowledge acquisition. However, it is essential to note that despite their impressive performance, LLMs such as Bard, GPT-3.5 and GPT-4 can sometimes generate text containing fabricated or incorrect information. This raises concerns about the credibility of information produced by LLMs, especially in educational settings where accuracy is crucial.

It is also important to acknowledge that LLMs, including GPT, come with both positive and negative consequences [37,38]. Friederichs et al [34] argue that the ability to acquire knowledge is a basic determinant of a physician’s performance, and GPT-3.5 should be looked upon as a tool that provides easy access to a lot of relevant information, eventually aiding in clinical decision-making processes. On the other hand, Mbakwe et al [39] have commented that GPT-3.5’s success on exams such as the USMLE demonstrates the flaws of medical education, which is “mostly focused on the rote memorization of mechanistic models of health and disease” and does not reward critical thinking to the same extent.

Further research and development are warranted to address the limitations and challenges posed by LLMs and ensure their reliable and accurate use in education and other domains. Moreover, future investigations can explore the suitability of LLMs for addressing the needs of diverse professional fields beyond medical entrance exams.

In conclusion, this study contributes valuable insights into the capabilities of Bard, GPT-3.5, and GPT-4 in handling high-stakes examination questions. As LLMs continue to evolve, their potential to revolutionize education and other industries remains promising, albeit with the need for continuous improvements and validation of their accuracy and reliability.

## Abbreviations

AI: artificial intelligence
FP: false positive
GPT: Generative Pre-trained Transformers
LLM: large language model
NEET: National Eligibility cum Entrance Test
NTA: National Testing Agency
TP: true positive
USMLE: United States Medical Licensing Examination

## Data Availability

Data can be obtained through a reasonable request to the corresponding author.
